# Effectiveness of Therapeutic Interventions in the Treatment of Internet Gaming Disorder: A Systematic Review

**DOI:** 10.3390/ejihpe15040049

**Published:** 2025-04-01

**Authors:** Sandra Núñez-Rodríguez, David Burgos-González, Luis Alberto Mínguez-Mínguez, Félix Menéndez-Vega, José Luis Antoñanzas-Laborda, Jerónimo Javier González-Bernal, Josefa González-Santos

**Affiliations:** 1 Department of Health Sciences, University of Burgos, 09001 Burgos, Spain; snr1005@alu.ubu.es (S.N.-R.); dbg0014@alu.ubu.es (D.B.-G.); laminguez@ubu.es (L.A.M.-M.); jejavier@ubu.es (J.J.G.-B.); mjgonzalez@ubu.es (J.G.-S.); 2 Area of Developmental and Educational Psychology, University of Zaragoza, 50009 Zaragoza, Spain; jlantona@unizar.es

**Keywords:** internet gaming disorder, intervention, children, adolescents, young adults, systematic review

## Abstract

Internet Gaming Disorder (IGD) has been recognized by the World Health Organization (WHO) in the International Classification of Diseases (ICD-11) and as an emerging condition in the DSM-5. IGD is increasingly prevalent, with various negative effects on individuals’ development and adaptation. To address this issue, different therapeutic interventions, like CBT, virtual reality, mindfulness, or family therapy, have been explored. This systematic review aimed to answer the following research question: What is the effectiveness of therapeutic interventions in reducing IGD symptoms in adolescents and young adults diagnosed with this disorder? Following PRISMA guidelines, 22 studies published between 2014 and 2025 were included. Results show that cognitive behavioral therapy (CBT) is the most effective intervention, significantly reducing IGD severity, anxiety, and depression. Combining CBT with physical exercise or mindfulness further enhanced outcomes. Other promising approaches include virtual reality (VR), transcranial direct current stimulation (tDCS), and family-based interventions. Additionally, treatments involving mindfulness and animal-assisted therapy showed potential in improving emotional regulation and interpersonal relationships. However, further research is needed to evaluate long-term efficacy and explore emerging therapies.

## 1. Introduction

Internet Gaming Disorder (IGD) has been recognized as a significant public health concern due to its impact on psychological, social, and functional well-being. The World Health Organization (WHO) included IGD in the International Classification of Diseases (ICD-11) and the Diagnostic and Statistical Manual of Mental Disorders (DSM-5) classified it as an emerging condition requiring further study (Castro-Calvo et al., 2021; Yen et al., 2022). IGD is characterized by impaired control over gaming, prioritization of gaming over other activities, and significant negative consequences in daily life, including academic, occupational, and social impairments (Auerbach et al., 2018; Jo et al., 2019).

The technological revolution and widespread access to online gaming have contributed to an increase in IGD cases, particularly among adolescents and young adults (Feng et al., 2017; Paulus et al., 2018). Several risk factors have been associated with IGD, including low self-esteem, difficulties in interpersonal relationships, high levels of stress, anxiety, and depression. In addition, lack of parental supervision, academic difficulties, and a history of other addictive behaviors increase the vulnerability to developing this disorder (Balhara et al., 2021; Fumero et al., 2020; Nielsen et al., 2020; Teng et al., 2020).

The most frequently reported symptoms of IGD include mood disturbances(depression, anhedonia, and anxiety). increased stress levels, low life satisfaction, and impulsivity (Bargeron & Hormes, 2017; De Pasquale et al., 2020; A. M. S. Wu et al., 2018). Given the growing prevalence of IGD and its negative impact, research has focused on (1) understanding its underlying mechanisms and risk factors and (2) evaluating the effectiveness of various therapeutic interventions (Lee et al., 2021; Pearcy et al., 2017).

Among these interventions, cognitive behavioral therapy (CBT) has been widely recognized as the first-line treatment for IGD, given its effectiveness in modifying addictive behaviors and maladaptive thought patterns (Dong et al., 2024; X. Han et al., 2018). However, recent studies have explored alternative and complementary interventions, such as mindfulness-based therapies, virtual reality (VR), transcranial direct current stimulation (tDCS), and family-based interventions (Li et al., 2017; Moghaddas et al., 2023; Park et al., 2016). These emerging approaches aim to enhance emotional regulation, improve impulse control, and strengthen social and family relationships.

### Aim and Research Question

This systematic review aims to evaluate the effectiveness of IGD treatments of different therapeutic interventions in reducing IGD symptoms among adolescents and young adults. Specifically, this review seeks to answer the following research question: What is the effectiveness of therapeutic interventions in reducing IGD symptoms in adolescents and young adults diagnosed with this disorder? This study was conducted following PRISMA guidelines, ensuring a rigorous methodology for selecting and analyzing the relevant literature.

A systematic search was performed in Web of Science, ScienceDirect, PubMed, and Scopus, using Medical Subject Headings (MeSH) and Boolean operators to optimize the selection of studies published between 2014 and 2025. Inclusion criteria focused on experimental and quasi-experimental studies that assessed the effectiveness of interventions specifically targeting IGD. Studies with low methodological quality, case reports, and research focusing on populations with comorbidities unrelated to IGD were excluded.

By synthesizing current evidence on IGD treatments, this review aims to provide a comprehensive evaluation of their efficacy, highlight their strengths and limitations, and suggest future directions for research and clinical practice.

## 2. Materials and Methods

Following the guidelines established by the PRISMA Declaration and in accordance with the defined research protocol, a systematic review of the scientific literature was conducted from 3 to 9 January 2025. To achieve this, the electronic versions of the Web of Science, ScienceDirect, PubMed, and Scopus databases were consulted.

The search was based on a clinically answerable research question using the PIO format (Table 1):

Subsequently, search strategies were designed and adapted for each database (Table 2). During the search process, the corresponding Medical Subject Headings (MeSH) were used, along with Boolean operators and free-text search terms, some of which were truncated to include all possible variations and word endings.

The systematic review included original research studies that met the following criteria: experimental or quasi-experimental design, publication in English or Spanish, publication date from 2014 onwards, availability of at least an abstract, and evaluation of the effectiveness of the intervention in IGD as part of the results. The selection of 2014 as the starting year is due to the fact that this was when the scientific community began to develop specific treatments for Internet Gaming Disorder following its inclusion in the DSM-5 as an emerging condition.

Case reports, scientific letters, low-quality records, and studies that did not answer the research question, were unrelated to the objective of the review, or focused on specific population subgroups under 10 years old or over 30 years old were excluded.

Additionally, although ADHD and MDD are major comorbidities of IGD, the objective of this review is to determine the effectiveness of different treatments in patients with IGD. Therefore, the selection of studies was limited to patients with IGD without comorbid disorders.

The selection of studies and the evaluation of their methodological quality were carried out in pairs, independently, and in a blinded manner. Any discrepancies between reviewers were resolved by consensus, and in cases of persistent disagreement, a third reviewer was consulted.

To ensure uniformity in data collection, a standardized data extraction form was designed, including the following aspects for each article: title and main author, country and year of publication, study type and objective, sample size and characteristics, variables and instruments used, a summary of the main findings and conclusions, as well as the results of methodological quality assessment using the critical appraisal tools of the Joanna Briggs Institute at the University of Adelaide (Australia), adapted to the design of each study [insert annexes with questions].

To include studies in the systematic review, a minimum threshold was established: 6 out of 9 points for quasi-experimental studies and 9 out of 13 points for experimental studies.

Prior to the final analysis, a pilot test was conducted in which each reviewer evaluated three articles, and the concordance between their assessments was subsequently compared.

## 3. Results

**Figure 1:**
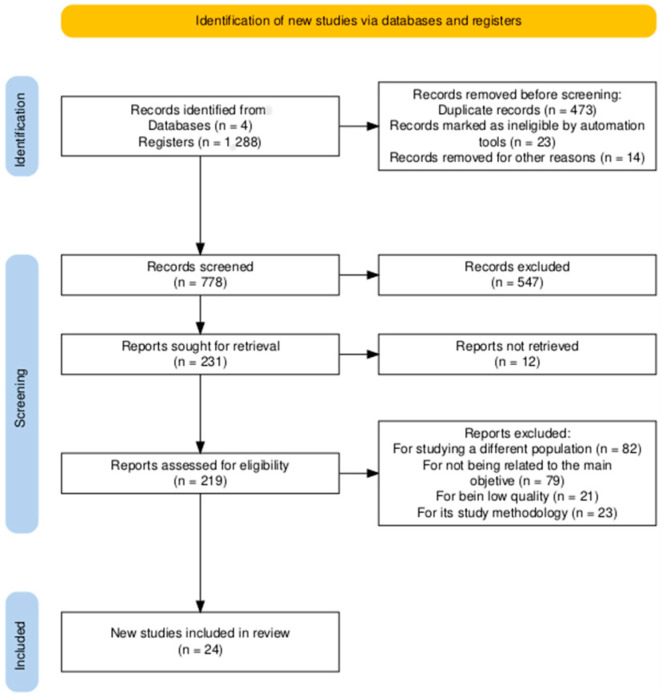
Flow-diagram for study selection.

Initially, 1288 studies were identified through the search strategies. After a full-text critical review, 24 studies were selected for inclusion in the systematic review (Figure 1).

With the selected studies, the previously mentioned data collection protocol was completed. The characteristics of the studies included in the systematic review can be found in Appendix A.

### 3.1. Description of the Characteristics of the Studies

A total of 24 studies were included in the systematic review, evaluating the effectiveness of different interventions for the treatment of Internet Gaming Disorder (IGD). Regarding study design, 11 studies were randomized clinical trials (RCTs), while 13 were quasi-experimental. The studies were conducted in Asia (n = 12, 50%), Europe (n = 6, 25%), North America (n = 4, 17%), and Oceania (n = 2, 8%), reflecting a global interest in IGD research.

The sample size ranged between 10 and 205 participants, focusing on adolescents and young adults aged 12 to 30 years. In more than half of the studies, participants were exclusively male (n = 12), while other studies included mixed samples (n = 9) with lower female representation, and one study did not specify the sex of the sample.

The instruments used assessed the severity of IGD with validated scales such as the Chen Internet Addiction Scale—CIAS (n = 4), Internet Addiction Test—IAT (n = 4), Internet Gaming Disorder Scale in both its long and short version (n = 5), Young Internet Addiction Scale (n = 3), DSM-5 diagnostic criteria (n = 3), Game Addiction Screening Assessment—GASA (n = 1), Game Addiction Screening Test (n = 1), or hours of gaming per day/week (n = 3). In addition, game craving was measured in nine of the selected studies, with instruments such as the Visual Analog Scale (n = 4), Signal Reactivity Task (fMRI) (n = 2), adaptation of the Tiffani questionnaire (n = 1), adaptation of the QSU-Brief smoking urgency questionnaire (n = 1), and the Online Game Signal Exposure Craving Questionnaire (n = 1).

To measure anxious symptoms (n = 12), the BAI scale was mostly used (n = 7), followed by the DASS-21 (n = 2) and the PSS-10 (n = 1). Depressive symptoms were measured in 11 studies, with the BDI as the most commonly used instrument (n = 8), together with the CDI (n = 1) and the DASS-21 (n = 2). Impulsivity was tested in six studies, mainly with the BIS-11 (three times) and the BIS/BAS (one time), while impulsivity in decision-making was assessed in five studies using the DDT (two times), the BART (two times) and the STT (one time). Brain activity and physiological responses were measured in nine studies, with fMRI (five times), EEG (two times), ECG (one time), and SCR (one time).

In addition, other variables such as personal and personality factors, quality of life and life satisfaction, interpersonal skills, physical activity and functional capacity, cognitive and general functioning, attachment, and family relationships were measured.

Of the 11 randomized controlled trials (RCTs) included in the review, five used a passive control group, meaning participants were placed on a waiting list or received no intervention (Hong et al., 2020; J. Han et al., 2020; Park et al., 2016; Maden et al., 2022; Zhao et al., 2022). In these studies, active treatments (such as cognitive behavioral therapy [CBT] or mindfulness-based therapy) showed significant improvements in reducing IGD severity, anxiety, and depression compared to no intervention. On the other hand, six studies included an active control group, where the experimental intervention was compared to an alternative therapeutic strategy, such as physical training, virtual reality, or transcranial direct current stimulation (tDCS) (Ni et al., 2024; Yao et al., 2017; Nielsen et al., 2021; Shin et al., 2021; Zheng et al., 2022; Kochuchakkalackal Kuriala & Reyes, 2023). Studies with an active control group provide greater incremental validity, allowing researchers to assess whether the benefits of a specific intervention exceed those of other available alternatives. In this regard, combined interventions, such as CBT plus physical exercise or mindfulness, demonstrated the most robust and sustained effects over time compared to isolated treatments.

#### Differentiation Between Clinical and Subclinical Samples

The studies included in this review varied in terms of sample characteristics, specifically whether participants met the full diagnostic criteria for Internet Gaming Disorder (IGD) or exhibited subthreshold symptoms. Of the 24 studies analyzed, 15 explicitly recruited participants diagnosed with IGD according to standardized criteria such as the DSM-5 or ICD-11 (J. Han et al., 2020; Hong et al., 2020; Nielsen et al., 2021; Park et al., 2016; Shin et al., 2021; Torres-Rodríguez et al., 2018; L. Wu et al., 2021; Zhang et al., 2016; Zhao et al., 2022; Zheng et al., 2022; André et al., 2023; Pornnoppadol et al., 2020; Kang et al., 2018; Marcelle et al., 2019; Sakuma et al., 2017). These studies primarily assessed the effectiveness of treatments in individuals with a confirmed clinical condition.

The remaining nine studies included participants with problematic gaming behaviors who did not necessarily meet the full diagnostic threshold for IGD but exhibited significant symptoms such as excessive gaming time, loss of control, and negative consequences in daily life (Igarashi et al., 2024; Kochuchakkalackal Kuriala & Reyes, 2023; Li et al., 2018; Maden et al., 2022; Nielsen et al., 2021; Ni et al., 2024; Zhang et al., 2018; L. Wu et al., 2021; Yao et al., 2017). Differentiating between clinical and subclinical samples is essential, as treatment effectiveness may vary depending on the severity of the disorder. It was observed that interventions such as cognitive behavioral therapy (CBT) and mindfulness-based therapy were effective in both groups; however, studies with clinical samples reported greater symptom reductions and more sustained effects over time.

As for statistical analyses, most used univariate tests to compare means before and after the intervention and multivariate tests to analyze different variables simultaneously.

When analyzing methodological quality and risk of bias, most studies achieved high scores, consistently exceeding the established threshold. The results can be seen in Table 3 and Table 4.

### 3.2. Description of the Results

#### 3.2.1. Internet Gambling Disorder

All the studies included in this review (n = 22) reported a significant reduction in IGD severity following intervention (André et al., 2023; J. Han et al., 2020; Hong et al., 2020; Igarashi et al., 2024; Kang et al., 2018; Kochuchakkalackal Kuriala & Reyes, 2023; Li et al., 2018; Maden et al., 2022; Marcelle et al., 2019; Ni et al., 2024; Nielsen et al., 2021; Park et al., 2016; Pornnoppadol et al., 2020; Sakuma et al., 2017; Shin et al., 2021; Torres-Rodríguez et al., 2018; L. Wu et al., 2021; Yao et al., 2017; Zhang et al., 2016, 2018; Zhao et al., 2022; Zheng et al., 2022). Within these, cognitive behavioral therapy stood out as the strategy with the most empirical support, significantly improving IGD symptomatology and in some cases reducing the severity of the disorder by up to 66.3% (André et al., 2023; J. Han et al., 2020; Hong et al., 2020; Kochuchakkalackal Kuriala & Reyes, 2023; Torres-Rodríguez et al., 2018; Zhang et al., 2016, 2018; Zheng et al., 2022).

In addition to CBT, other approaches have been studied with positive results. Three studies evaluated mindfulness-based therapy. This strategy showed a significant reduction in IGD symptoms, especially in emotional regulation and impulse control (Li et al., 2018; Ni et al., 2024; Yao et al., 2017). On the other hand, three studies investigated the efficacy of virtual reality (VR) therapy, reducing gaming time, and significantly improving control over gaming impulses after the intervention. Thus, research is beginning to suggest that immersion in controlled VR environments may facilitate awareness of the negative effects of gambling. (Maden et al., 2022; Park et al., 2016; Shin et al., 2021).

The most effective results have been found when several interventions are combined (Hong et al., 2020; Pornnoppadol et al., 2020; Yao et al., 2017; Zheng et al., 2022). For example, the combination of CBT with physical exercise was highlighted as having the most pronounced decrease in symptoms compared to isolated interventions (Hong et al., 2020). Also, the addition of CBT with meditation and relaxation techniques significantly decreased impulsivity and weekly play time (Yao et al., 2017).

Other interventions such as the use of transcranial direct current stimulation (tDCS) (L. Wu et al., 2021) and Multidimensional Family Therapy (MDFT) (Nielsen et al., 2021) have been explored and have shown promising results. One study indicated that tDCS applied to the dorsolateral prefrontal cortex significantly reduced gambling craving and improved self-control in individuals with IGD (L. Wu et al., 2021). In the case of MDFT, it substantially reduced IGD symptomatology and improved family cohesion (Nielsen et al., 2021).

#### 3.2.2. Treatment Effectiveness in Reducing Anxious and Depressive Symptoms

The efficacy of interventions on anxiety and depressive symptoms were studied in 11 of the selected studies (J. Han et al., 2020; Hong et al., 2020; Kang et al., 2018; Maden et al., 2022; Marcelle et al., 2019; Park et al., 2016; Yao et al., 2017; Zhang et al., 2016, 2018; Zhao et al., 2022; Zheng et al., 2022). In relation to anxiety, seven studies using the BAI to assess the evolution of symptoms concluded a significant reduction in anxiety (J. Han et al., 2020; Hong et al., 2020; Maden et al., 2022; Park et al., 2016; Zhang et al., 2016, 2018; Zhao et al., 2022). In particular, interventions involving CBT and mindfulness techniques were found to be more effective, improving emotional regulation and helping to decrease levels of physiological arousal associated with impulsivity and gambling cravings (Yao et al., 2017).

Regarding depression, seven studies used the BDI and also showed significant improvements in the reduction in depressive symptoms associated with IGD (J. Han et al., 2020; Hong et al., 2020; Park et al., 2016; Yao et al., 2017; Zhang et al., 2016, 2018; Zhao et al., 2022). The best evaluated technique was individual and/or group CBT, which helped patients to change negative thoughts related to feelings of lack of control and self-image. In addition, equine-assisted therapy also reported significant results on anxious–depressive symptomatology (Kang et al., 2018).

Again, combined interventions have shown better results. The combination of CBT with physical exercise achieved the greatest reduction in anxiety and depression levels compared to the isolated use of the technique (Hong et al., 2020). On the other hand, CBT combined with mindfulness has also resulted in a more marked decrease in anxious–depressive symptomatology (Yao et al., 2017).

On the other hand, VR approaches found a reduction in gambling anxiety, just as tDCS was effective in reducing depressive symptoms (Igarashi et al., 2024; Maden et al., 2022; Shin et al., 2021).

Finally, five studies looked at impulsiveness in decision-making, finding that the combined intervention of reality therapy together with mindfulness significantly reduced intertemporal impulsivity. Other studies showed that training inhibitory control and reward sensitivity significantly reduced this condition (Park et al., 2016; Sakuma et al., 2017; Yao et al., 2017; Zhang et al., 2016; Zheng et al., 2022).

#### 3.2.3. Effectiveness of Treatment in Improving Interpersonal Relationships and Family Functioning

Four studies of the 22 included in the review provided results on the effectiveness of their interventions on the family functioning and interpersonal skills of affected individuals (J. Han et al., 2020; Nielsen et al., 2021; Sakuma et al., 2017; Torres-Rodríguez et al., 2018). Interventions include structured family therapy, specialized social skills training programs, and VR.

In this context, Multidimensional Family Therapy (MDFT) proved to be the most effective strategy in improving the family environment (Nielsen et al., 2021). Moreover, participants improved their quality of life, their family relationships and decreased the severity of IGD more than those who received regular family therapy.

On the other hand, PIPATIC demonstrated positive effects on the improvement of interpersonal relationships. This program combines CBT with social skills training, and significantly increased family cohesion as well as the quality of personal interactions. In addition, the program also improved communication skills, emotional regulation and conflict resolution skills (Torres-Rodríguez et al., 2018).

In addition to interventions involving family members, other individual interventions with affected individuals also improved interpersonal relationships. For example, VR interventions reduced parents’ perceived conflict intensity and increased control of emotional responses to family stressful situations after training in a controlled digital environment on problem-solving practices for IGD-triggered family conflicts (Nielsen et al., 2021; Shin et al., 2021).

On the other hand, an intervention based on a stay in a residential therapeutic camp showed that being in a structured, technology-free social environment facilitated participants’ development of social skills and increased the perception of social support among them (Sakuma et al., 2017).

#### 3.2.4. Changes in Brain Connectivity and Neurophysiology

A total of seven studies in studies included in the review examined the effects of interventions on brain activity, using measurement tools such as fMRI, EGG, and ECG (Hong et al., 2020; Kang et al., 2018; Ni et al., 2024; Park et al., 2016; Zhang et al., 2016, 2018; Zhao et al., 2022).

Therapeutic strategies evaluated included mindfulness-based interventions and CBT, which were effective in modifying brain connectivity (Ni et al., 2024). Studies indicated a significant increase in functional connectivity in the ventral striatum, medial frontal gyrus, and insula following the application of these therapies, regions responsible for reward processing, emotional regulation, and self-control (Hong et al., 2020; Kang et al., 2018; Ni et al., 2024; Park et al., 2016; Zhang et al., 2016, 2018; Zhao et al., 2022).

In addition, physical exercise increased frontal alpha asymmetry, a neural biomarker related to emotional balance and stress reduction (Hong et al., 2020)). Furthermore, tDCS on the dorsolateral prefrontal cortex significantly reduced the activation of circuits associated with reactivity to gambling cues, which improves inhibitory control and reduces impulsivity (L. Wu et al., 2021).

Additionally, animal-assisted therapy produced greater activation in the medial prefrontal cortex and the limbic system, which are responsible for emotional regulation and the processing of interpersonal relationships (Kang et al., 2018).

#### 3.2.5. Differential Outcomes Based in Follow-Up

Analysis of follow-up data revealed differential outcomes based on the type of intervention and the duration of post-treatment assessment. Studies utilizing CBT and mindfulness-based therapies reported the most stable long-term results, with symptom reductions maintained up to 6–12 months post-intervention (André et al., 2023; Hong et al., 2020; Yao et al., 2017). In contrast, interventions based on neuromodulation (tDCS) and VR tended to show short-term improvements, with a gradual decline in effectiveness observed after 3–6 months, particularly in the absence of reinforcement strategies (Park et al., 2016; Shin et al., 2021). Additionally, studies that incorporated family-based interventions or structured relapse prevention plans showed greater treatment adherence and lower relapse rates, reinforcing the importance of multimodal approaches that integrate psychological, neurophysiological, and social support strategies.

To summarize and better understand each study reviewed, please refer to Appendix A, where the main characteristics of the studies and their key findings are included.

## 4. Discussion

The aim of this review was to analyze the existing literature on the effectiveness of treatments for IGD. During the review, it has been possible to analyze how different interventions influence the severity of the disorder, the most frequent comorbid symptomatology, such as anxiety and depression, change in family and interpersonal relationships, and the neurophysiological alterations associated with IGD. The findings reinforce the importance of structured, evidence-based treatments, particularly cognitive behavioral therapy (CBT), while also highlighting the potential benefits of alternative and combined interventions.

In terms of sex distribution, IGD predominantly affects males, as observed in most of the analyzed studies, which included predominantly male samples (Hong et al., 2020; Kang et al., 2018; Maden et al., 2022; Park et al., 2016; Sakuma et al., 2017; Shin et al., 2021; Torres-Rodríguez et al., 2018; L. Wu et al., 2021; Zhang et al., 2016, 2018; Zheng et al., 2022). Even in mixed-gender studies, male participants significantly outnumbered females (André et al., 2023; Igarashi et al., 2024; Kochuchakkalackal Kuriala & Reyes, 2023; Li et al., 2018; Marcelle et al., 2019; Ni et al., 2024; Nielsen et al., 2021; Pornnoppadol et al., 2020; Zhao et al., 2022).

Psychological interventions, particularly CBT, have been identified as the most effective treatment for IGD, significantly reducing disorder severity and associated symptoms (André et al., 2023; J. Han et al., 2020; Hong et al., 2020; Kochuchakkalackal Kuriala & Reyes, 2023; Torres-Rodríguez et al., 2018; Zhang et al., 2016, 2018; Zheng et al., 2022). CBT helps individuals develop self-regulation skills, restructure maladaptive thoughts, and implement behavioral changes aimed at reducing gaming dependence. However, findings suggest that combined interventions may enhance long-term treatment outcomes, particularly those integrating CBT with physical activity or mindfulness-based therapy (Hong et al., 2020; Pornnoppadol et al., 2020; Yao et al., 2017; Zheng et al., 2022).

Alternative interventions such as virtual reality therapy (VR), transcranial direct current stimulation (tDCS), and animal-assisted therapy have also demonstrated effectiveness in reducing IGD symptoms. VR-based interventions appear to enhance self-awareness and impulse control, while tDCS may improve cognitive flexibility and reduce compulsive gaming urges. Moreover, recent studies indicate that neurofeedback training and serious gaming interventions may be promising complementary strategies, helping individuals develop greater impulse control by providing real-time feedback on brain activity and reinforcing positive behavioral changes. However, the limited number of supporting studies, small sample sizes, and lack of long-term follow-up data restrict conclusions about their sustained effectiveness (Kang et al., 2018; Maden et al., 2022; Nielsen et al., 2021; Park et al., 2016; Shin et al., 2021; L. Wu et al., 2021). Future research should include larger trials with extended follow-up periods to evaluate their sustained impact and compare their efficacy to more established interventions. IGD is frequently associated with anxious and depressive symptomatology, reinforcing the need for treatments that address both gaming behavior and underlying psychological distress (Bargeron & Hormes, 2017; De Pasquale et al., 2020; Maden et al., 2022; Marcelle et al., 2019). In this regard, the studies reviewed suggest that integrative approaches combining CBT with mindfulness and physical exercise show the most promising results (Hong et al., 2020; Maden et al., 2022). These results emphasize CBT as the most empirically supported treatment, increasing its efficacy if combined with physical activity, since it has been shown to improve executive function and promote the release of neurotransmitters related to emotional well-being, which potentiates the effects of CBT, as well as with mindfulness, probably due to the strengthening of emotional self-regulation and the reduction in ruminative thinking (Li et al., 2018; Magill et al., 2019; Pascoe et al., 2020; Smith & Merwin, 2021; Yao et al., 2017; Zheng et al., 2024). Additionally, research suggests that individuals with IGD often present cognitive distortions related to gaming as a coping mechanism for emotional distress, which may explain the strong association between IGD and depression. These distortions include overestimation of gaming skills, avoidance of real-life responsibilities, and reliance on gaming for emotional regulation. Addressing these factors through cognitive restructuring techniques within CBT could further enhance treatment outcomes. Given the strong correlation between IGD and depression, these findings underscore the importance of screening for depressive symptoms in IGD patients and incorporating interventions that target both conditions simultaneously.

On the other hand, this disorder affects all spheres of life, also having a negative impact on interpersonal relationships and family functioning. People with IGD often have impaired communication with their families and friends, increased family conflicts and difficulties in establishing social relationships outside the digital environment (Fumero et al., 2020; Teng et al., 2020). The studies included in the review highlight the value of family involvement in the recovery process, particularly in adolescent populations. Family-based interventions such as Multidimensional Family Therapy (MDFT) and the PIPATIC program have demonstrated significant improvements in family dynamics, communication, and the establishment of healthy gaming limits, which are key factors in preventing relapse and fostering long-term recovery (Nielsen et al., 2021; Torres-Rodríguez et al., 2018). In addition, VR-based and social skills training interventions have shown effectiveness in reducing family conflict and improving interpersonal cohesion (Shin et al., 2021).

Beyond structured family-based interventions, studies have indicated that parents’ digital literacy and gaming-related attitudes play a crucial role in the success of IGD interventions. Parents who actively engage in digital education programs and set consistent gaming-related boundaries tend to have adolescents with better treatment adherence and lower relapse rates. Therefore, integrating parental education components into IGD interventions could further enhance long-term treatment effectiveness.

Finally, the literature supports that IGD is associated with alterations in brain activity, in particular in regions related to impulse control and emotional regulation. Neuroimaging studies indicate that IGD is associated with altered neural activity in regions responsible for impulse control, reward processing, and emotional regulation. For its part, interventions for IGD may therefore induce changes in neural connectivity and physiology in patients (Hong et al., 2020; Kang et al., 2018; Ni et al., 2024; Park et al., 2016; Zhang et al., 2016, 2018; Zhao et al., 2022). The reviewed studies show that combined interventions of CBT with physical exercise or mindfulness, as well as animal therapy and tDCS, strengthen the activity of the ventral striatum, prefrontal cortex and insula, reducing gambling craving, and increasing self-control and emotional regulation (Kang et al., 2018; L. Wu et al., 2021; Yao et al., 2017; Zheng et al., 2022).

Additionally, emerging research suggests that abnormalities in dopamine regulation and prefrontal cortex function may contribute to the compulsive nature of IGD. Studies using functional MRI have shown that individuals with IGD exhibit increased activity in reward-processing regions (such as the striatum) and reduced activity in areas associated with cognitive control (such as the dorsolateral prefrontal cortex) (Kang et al., 2018; L. Wu et al., 2021; Yao et al., 2017; Zheng et al., 2022). These findings suggest that targeting neurobiological mechanisms through pharmacological or neuromodulation approaches may enhance treatment outcomes, particularly for individuals with severe IGD who do not respond to conventional psychotherapy.

### Limitations and Future Research Directions

This review provides information that will guide future research and facilitate the development of programs to reduce IGD and/or alleviate symptoms derived from the disorder. Knowledge of IGD continues to advance in terms of understanding it and how to intervene with this new problem that is advancing not only in terms of severity, but also in terms of the population it affects. On the one hand, the adolescent population that has grown up with the unlimited use of screens is growing, and with it, the age group suffering from this disorder is increasing. The new generations have an increasingly uncontrolled use of technology from a very early age, making them a population at risk from childhood.

For future research, these results should be interpreted taking into account the limitations of the review, such as small sample sizes and limited follow-up periods. This reduces the possibility of inferring long-term therapeutic effects. Future lines of research should explore the impact of IGD on academic performance and its potential relationship with school dropout rates, as this could provide valuable insights into the broader effects of the disorder on adolescent development. On the other hand, more studies directly comparing the efficacy of different therapeutic approaches would be interesting, as well as studies with emerging interventions that can be compared with CBT. In addition, due to the correlation seen in this review of the disorder with depression, future research and/or reviews could focus on this.

## 5. Conclusions

The results of this review suggest that IGD is a multidimensional disorder that requires comprehensive therapies addressing various areas of the individual. CBT is the most empirically supported intervention due to its effectiveness and also due to the long list of research that has been carried out on this technique. Moreover, its effectiveness can be enhanced by combining it with techniques such as physical exercise, mindfulness, or VR.

On the other hand, the integration of family and social relationships in the therapeutic process is important to improve the functionality of patients, as well as to improve their quality of life.

In future research, it is interesting to conduct studies with prolonged follow-ups to evaluate long-term interventions, as well as to combine therapeutic approaches and the use of emerging intervention tools.

## Figures and Tables

**Table 1:** PIO format.

Population	Adolescents and Young Adults (10–30 Years Old) Diagnosed with Gaming Disorder
Intervention	Treatment strategies, therapeutic interventions
Outcomes	Reduction in IGD symptoms
Research Question	What is the effectiveness of therapeutic interventions in reducing IGD symptoms in adolescents and young adults diagnosed with this disorder?

**Table 2:** Search strategies.

Database	Search Strategy
Pubmed	(“Gaming Disorder” OR “Video Game Addiction” OR “Internet Gaming Disorder” OR “Problematic Gaming” OR “Gaming Dependency” OR “Video Game Overuse” OR “Excessive Gaming”) AND (“adolescents” OR “young adults” OR “children” OR “teenagers” OR “youth”) AND (“therapy” OR “treatment” OR “psychological intervention” OR “cognitive therapy” OR “behavioral therapy” OR “mental health therapy” OR “CBT” OR “cognitive behavioral therapy”) AND (“outcomes” OR “symptoms” OR “improvement” OR “reduction” OR “health improvement” OR “clinical improvement”)
Web of Science	TS = (“Gaming Disorder” OR “IGD” OE “Video Game Addiction” OR “Internet Gaming Disorder” OR “Problematic Gaming” OR “Gaming Dependency” OR “Video Game Overuse” OR “Excessive Gaming”) AND TS = (“therapy” OR “treatment” OR “psychological intervention” OR “cognitive therapy” OR “behavioral therapy” OR “CBT” OR “cognitive behavioral therapy” OR “mental health therapy”) AND TS = (“symptoms” OR “outcomes” OR “improvement” OR “reduction” OR “health improvement” OR “clinical improvement”)
Scopus	TITLE-ABS-KEY(“Gaming Disorder” OR “Video Game Addiction” OR “Internet Gaming Disorder” OR “Problematic Video Gaming” OR “Gaming Dependency” OR “Excessive Gaming”) AND TITLE-ABS-KEY(“adolescents” OR “young adults” OR “children” OR “teenagers” OR “youth”) AND TITLE-ABS-KEY(“therapy” OR “treatment” OR “intervention” OR “psychological therapy” OR “behavioral therapy” OR “CBT”) AND TITLE-ABS-KEY(“symptoms” OR “outcomes” OR “improvement” OR “reduction”)
Science Direct	(“Gaming Disorder” OR “IGD” OR “Internet Gaming Disorder” OR “Video Game Addiction”) (“therapy” OR “treatment” OR “psychological intervention”) (“adolescents” OR “young adults” OR “youth”) (“symptoms” OR “outcomes” OR “clinical improvement”)

**Table 3:** Results of the quality assessment of randomized trials.

Study	Q1	Q2	Q3	Q4	Q5	Q6	Q7	Q8	Q9	Q10	Q11	Q12	Q13
(Ni et al., 2024)	+	+	+	-	-	-	+	+	+	+	+	+	+
(Hong et al., 2020)	+	+	+	-	-	-	+	+	+	+	+	+	+
(Park et al., 2016)	+	+	+	-	-	+	+	+	+	+	+	+	+
(Maden et al., 2022)													
(Zheng et al., 2022)	+	-	+	-	+	-	+	-	+	+	+	+	+
(Kochuchakkalackal Kuriala & Reyes, 2023)	+	-	+	+	-	-	+	+	+	+	+	+	+
(L. Wu et al., 2021)	+	+	+	+	+	+	+	-	-	+	+	+	+
(Nielsen et al., 2021)	+	+	+	-	-	+	+	+	+	+	+	+	+
(Zhao et al., 2022)	+	+	+	+	-	+	+	+	+	+	+	+	+

**Table 4:** Results of the quality assessment of quasi-experimental studies.

Study	Q1	Q2	Q3	Q4	Q5	Q6	Q7	Q8	Q9
(Shin et al., 2021)	+	+	+	+	+	-	+	+	+
(André et al., 2023)	+	+	+	+	+	+	+	+	+
(Torres-Rodríguez et al., 2018)	+	+	+	+	+	+	+	+	+
(Igarashi et al., 2024)	+	+	-	+	+	+	+	+	+
(Marcelle et al., 2019)	+	+	-	+	+	+	+	+	+
(Zhang et al., 2016)	+	+	-	+	+	+	+	+	+
(Pornnoppadol et al., 2020)	+	+	+	+	+	+	+	+	+
(Zhang et al., 2018)	+	+	-	+	+	+	+	+	+
(Kang et al., 2018)									
(J. Han et al., 2020)	+	+	+	+	+	+	+	+	+
(Li et al., 2018)	+	+	+	+	+	+	+	+	+
(Sakuma et al., 2017)	+	+	+	-	+	+	+	+	+
(Yao et al., 2017)	+	+	+	+	+	-	+	+	+

## Data Availability

Data are contained within the article.

## Appendix A. Characteristics of the Studies Included in the Systematic Review

**Study/Author**	**Typology/Main Objective**	**Participants**	**Variables/Instruments**	**Main Findings**	**JBI**
(Shin et al., 2021)	Design: Non-randomized experimental Objective: To examine the potential use of a virtual reality application in managing conflict related to games with young adults with TGI and matched controls.	N = 50 Sex: Male Average age: 21.78 (SD 2.33)	Intelligence Quotient (IQ): Wechsler Adult Intelligence Scale (revised) Internet gaming behavior: Frequency measured in hours of gameplay per week Gaming cravings, intensity of anger, coping skills: VAS Motivation to Change: Modified readiness to change questionnaire Virtual Reality System Experience: PQ and SSQ Tasks Behavioral Reactions: Initial expression of anger and choice to stop or continue playing Data recorded by the VR system: Response time, head movement, hand gestures, speech content, and conversation duration	There were no significant differences between the IGD and control groups in terms of age (*p* = 0.83), education (*p* = 0.11), or IQ (*p* = 0.36). The IGD group showed higher gaming cravings (*p* < 0.001), greater severity of the disorder (N/A), and more hours of gameplay per week (*p* < 0.001) compared to the control group. In the Presence Questionnaire (PQ), participants with IGD showed significantly lower scores than the controls (*p* < 0.001). The IGD group experienced higher levels of anger compared to the control group (*p* = 0.006). Furthermore, anger was higher when the stimulus was the virtual father compared to the virtual mother (*p* = 0.04) and increased in the second expression of anger compared to the first (*p* = 0.03). Participants in the control group perceived significantly more benefits than risks when stopping the game (*p* = 0.003). In the IGD group, a greater perception of benefits was associated with higher motivation to change their gaming behavior (r = 0.488, *p* = 0.01). The evaluation of benefits had a stronger effect than the evaluation of risks (*p* < 0.001), and the control group showed greater sensitivity to benefits than to risks compared to the IGD group (*p* < 0.001). The IGD group showed a higher tendency to continue playing compared to the control group (*p* < 0.001). However, both groups showed a greater willingness to stop playing after the risk–benefit evaluation compared to the start (*p* = 0.003) and after the expression of anger (*p* = 0.02). The control group showed a better understanding of the perspective of the virtual parents than the IGD group (*p* < 0.001), and the perspective of the virtual mother was better understood than that of the virtual father (*p* = 0.04). The control group perceived the coping strategy as more helpful in resolving conflicts compared to the IGD group (*p* = 0.004), but there were no significant differences in the perceived usefulness of anger expression (*p* = 0.71). In the control group, more hours of gameplay were associated with a greater perception of the usefulness of coping strategies over anger expression (r = 0.556, *p* = 0.003). In contrast, in the IGD group, fewer hours of gameplay were associated with a greater perception of coping strategies over anger expression (r = −0.468, *p* = 0.02).	8/9
(Ni et al., 2024)	Study type: Randomized clinical trial Objective: Explore the efficacy of mindfulness used to treat adults with IGD and identify the neural mechanisms underlying mindfulness.	N = 80 Sex: 33 women, 31 men Age: >18 years	Addiction: DSM-5-TR and Internet Addiction Test (IAT) Craving: Gaming Urge Questionnaire (Adaptation of Tiffany’s Questionnaire) Brain Responses: BOLD Signals Functional Brain Connectivity: CONN Toolbox, Version 22a, Regions of Interest (ROI)	The final analysis included a total of 64 adults with IGD, with 32 participants assigned to the Mindfulness (MM) group and 32 to the Progressive Muscle Relaxation (PMR) group. After the intervention, IGD severity decreased from 7 to 3.6 points in the MM group (*p* < 0.001) and from 7.1 to 6.0 points in the PMR group (*p* = 0.04). The IAT score significantly decreased in the MM group from 70.1 to 43.0 points (*p* < 0.001), whereas no significant changes were observed in the PMR group (69.3 to 69.2, *p* = 0.11). Gaming craving significantly decreased in the MM group from 58.8 to 33.6 points (*p* < 0.001), while no significant reduction was found in the PMR group. Neuroimaging results showed that the MM group exhibited a significant reduction in activity within the bilateral lentiform nuclei (associated with reward processing and habit formation) (*p* = 0.02), the left medial frontal gyrus (MFG) (involved in cognitive control and emotional regulation) (*p* = 0.01), the right insula (linked to emotion perception and self-awareness) (*p* = 0.047), and the right sublobar region (related to sensory and emotional integration). Additionally, the MM group showed a significant increase in connectivity between the left medial frontal gyrus and the left lentiform nucleus (*p* = 0.03).	10/13
(Hong et al., 2020)	Design: Randomized trial Objective: To evaluate the effect and neurophysiological mechanisms of a physical exercise + cognitive behavioral therapy (CBT) intervention in individuals with IGD.	N = 54 Age: 13–18 years Sex: Male Intervention Groups: - 27 CBT + Physical Exercise (PE) - 27 CBT	Internet Addiction: YIAS (Young Internet Addiction Scale) Depression: BDI (Beck Depression Inventory) Anxiety: BAI (Beck Anxiety Inventory) Hyperactivity/ADHD: K-ARS Frontal Brain Activity: EEG Physical Exercise: Heart Rate	Both interventions started under similar conditions. The TCC+EF group showed a significant reduction in BDI (*p* = 0.001), BAI (*p* < 0.05), K-ARS (*p* < 0.05), and YIAS (*p* < 0.001) scores. The TCC-only group achieved a significant reduction in BDI (*p* < 0.05) and YIAS (*p* = 0.001) scores. Improvements in BDI and YIAS scores were significantly greater in the TCC+EF group compared to the TCC-only group. Both interventions were effective, but the TCC+EF group demonstrated a greater impact. In the TCC+EF group, a significant increase in frontal alpha asymmetry (FAA) values at F4-F3 (*p* < 0.001) was observed, along with a trend-level increase in FAA at F8-F7 (*p* = 0.05). However, no significant changes were found in these values for the TCC-only group.	10/13
(Park et al., 2016)	Design: Randomized clinical trial Objective: Investigate the therapeutic efficacy of virtual reality therapy (VRT) for gaming addiction.	N: 36 Sex: Male Age: >18 Group Distribution: 12 CBT, 12 VRT, 12 occasional users	Game Addiction: YIAS Depression: BDI Anxiety: BAI ADHD: ASRS-K Brain Activity (fMRI): Philips Achieva 3.0 T. Scanner, DPARSFA, and SPM8	After the treatment, both groups (CBT *p* = 0.05 and VRT *p* < 0.01) showed a significant reduction in YIAS scores. No significant differences were found in YIAS score changes between the CBT and VRT groups (*p* = 0.52). Following the intervention, the VRT group showed a significant increase in connectivity from the PCC seed to the right parietal precuneus, right superior temporal gyrus, left middle frontal gyrus, and left temporal fusiform gyrus. This change may suggest improved brain integration and greater activation in areas related to attention, memory, and emotional control. In the CBT group, significant increased connectivity was observed from the PCC to the right cerebellum, bilateral thalamus, and left occipital lingual gyrus. These changes may be associated with improved cognitive skills and emotional regulation, which are key processes in cognitive behavioral therapy.	11/13
(Maden et al., 2022)	Design: Randomized controlled trial Objective: To compare the effects of virtual reality training (VRT) and aerobic training (AT) programs on gaming disorder severity, physical activity, physical fitness, and anxiety, compared to a control group.	N = 45 Sex: Male Age: 18–28 years	Gaming Addiction: IGDS9-SF Physical Activity/Sedentary Level: IPAQ Functional Capacity: 6-MWT Physical Fitness (Strength and Flexibility): CST, ACT, CSRT, BST Maximal Oxygen Consumption: 20-meter shuttle run test Anxiety: BAI Perceived exertion during exercise: RPE	There were no demographic differences between groups except for age: the VRT group was older than both the AT and control groups (*p* = 0.040). Both the VRT and AT groups significantly reduced gaming time (*p* = 0.04 and *p* = 0.039, respectively) and sedentary time (*p* = 0.000 and *p* = 0.025) compared to the control group. Both intervention groups showed a significant increase in weekly physical activity levels (*p* = 0.032 for VRT and *p* = 0.042 for AT) compared to the control group. There was a significant reduction in IGDS9-SF scores in the VRT and AT groups compared to the control group (*p* = 0.041 and *p* = 0.045, respectively). Both groups improved their physical fitness, with significant differences in the following: - Six-Minute Walk Test (6-MWT) distance: Improvements in the VRT (*p* = 0.002) and AT (*p* = 0.000) groups. - Predicted VO2 max: Significant increases in VRT (*p* = 0.041) and AT (*p* = 0.032). - Upper body muscle strength (ACT): Increases in both groups, with a greater effect in VRT (*p* = 0.000) compared to AT (*p* = 0.011). - The VRT group also showed improvements in lower limb flexibility (CSRT) and upper limb flexibility (BST). Anxiety levels (BAI) significantly decreased in the VRT (*p* = 0.040) and AT (*p* = 0.011) groups but not in the control group. In the physical fitness analysis (Senior Fitness Test parameters and VO2 max), CSRT significantly influenced BST within the control group (*p* = 0.004). However, no other significant interactions were observed in the MANOVA analysis. Both intervention programs (VRT and AT) proved effective in reducing gaming disorder severity, sedentary time, and anxiety levels while improving physical activity levels, physical fitness (6-MWT, ACT, VO2 max), and flexibility. However, the VRT group showed greater effects on limb flexibility. These findings suggest that physical activity-based interventions can be a valuable tool in the treatment of gaming disorder.	
(Zheng et al., 2022)	Design: Randomized controlled trial Objective: To investigate whether there were different effects of a single intervention targeting either impulsive action or reward sensitivity compared to a combined intervention in individuals with Internet Gaming Disorder.	N = 80 Age: ≈14.75 years Sex: Male	Online Gaming Addiction: OGAS Impulsivity: Barratt-11 Depression, Anxiety, and Stress: DASS-21 Gaming Craving: Gaming Cue Exposure Craving Questionnaire (developed by researchers) Behavioral Impulsivity: Stop-Signal Task (STT) Reward Sensitivity: Stimulus-Response Compatibility Task (SRC) Behavioral Training: Go/No-go Task and SRC Task	No significant differences were found between the groups in demographic characteristics or baseline-related indices (*p* > 0.05). The response inhibition (RI) training group and the combined training group showed significant reductions in reaction times (SSRT) in the post-test (*p* < 0.001), while the ApBM and control groups showed no significant changes. The ApBM group and the combined training group exhibited significant reductions in SRC scores in the post-test (*p* < 0.001), whereas the RI and control groups showed no significant changes. The combined training group demonstrated significant reductions in IGD scores in both the post-test and follow-up assessments (*p* < 0.01 and *p* < 0.001, respectively). The RI and ApBM groups showed marginally significant reductions at certain points (*p* = 0.065 and *p* = 0.054, respectively). The control group showed no significant differences between measurements. The combined training group showed significant reductions in depression scores in both the post-test and follow-up (*p* < 0.001), whereas the RI and ApBM groups had less consistent results. The control group showed no significant changes. There was a significant reduction in general anxiety across assessments (*p* < 0.001), but no significant interaction between groups and measurements. Regarding stress and craving, no significant effects were found (*p* > 0.05). Changes in impulsivity (SSRT) and stimulus–response compatibility (SRC) scores explained 12.4% of the variations in IGD scores (adjusted R^2^ = 0.124, *p* < 0.01). Both SSRT and SRC were significant predictors of IGD scores (*p* < 0.05).	9/13
(Kochuchakkalackal Kuriala & Reyes, 2023)	Design: Randomized controlled trial (RCT) Objective: To evaluate the effectiveness of a psychological intervention program (ACRIP) aimed at adolescents with Internet Gaming Disorder (IGD).	N = 30 Age: 16–19 years Sex: Male and female	Internet Gaming Disorder: IGDS9-SF Psychological Well-being: Ryff’s PWB	A significant reduction in IGD symptoms was observed after the intervention (t = 15.98, *p* = 0.001). A significant improvement in psychological well-being was also noted (t = −42.56, *p* = 0.001). The ACRIP intervention had a significant effect on both outcomes: reduction in IGD (F = 493.42) and improvement in psychological well-being (PWB) (F = 2210.54), with an effect size of 0.90. No significant differences were found between pre-test and post-test scores for IGD or PWB in the control group.	10/13
(André et al., 2023)	Design: Unblinded randomized controlled trial Objective: To determine the efficacy of cognitive behavioral therapy (CBT) as a treatment for individual conduct disorder and problematic gaming (IGD) in adolescents.	N = 102 Age: 13–18 years Sex: Male and female	Internet Gaming Disorder: GASA (Game Addiction Screening Assessment) Psychological Well-being: PWB	Both the control and treatment groups showed a reduction in GASA scores over time, but the improvement was significantly greater in the treatment group. In the treatment group, the mean score decreased by 9.9 points (*p* < 0.001), while in the control group, the reduction was 5.1 points (*p* < 0.001). Demographic variables (age, gender, living situation) and common diagnoses did not have a significant impact on the improvement. The proportion of problematic and addicted gamers significantly decreased in the treatment group at follow-up compared to baseline. However, there were no significant differences in the prevalence of player categories in the control group between baseline and follow-up.	
(Torres-Rodríguez et al., 2018)	Study Design: Non-randomized experimental study Objective: To evaluate the effectiveness of a specialized psychotherapy program for adolescents with Internet Gaming Disorder (IGD), specifically the Individualized Psychotherapeutic Program for Addiction to Information and Communication Technologies (PIPATIC).	N = 31 Age: 12–18 years Sex: Male	Internet Gaming Disorder: IGD-20 Test Comorbid Disorders: YSR/11-18 and CBCL/6-18 Intrapersonal Concerns: MACI Emotional Intelligence: TMMS-24 (Trait Meta-Mood Scale) Interpersonal Skills: Social Skills Scale (EHS) Family Relationships: Family Discord G Clinical Severity: CGI-SI and CGI-GI General Functioning: GAF (Global Assessment of Functioning)	Before the treatment, no significant differences were observed between the experimental group (EG) and the control group (CG), with a *p*-value of 0.384. The EG showed a significant reduction in weekly gaming time (11.15 hours), whereas the CG did not experience a significant change (31.80 hours), with *p* = 0.0001. The EG significantly reduced delay to 1.81, while the CG maintained a higher value (3.27), with a significant difference (*p* = 0.0001). The EG experienced a significant reduction in subjective addiction (3.13) compared to the CG (6.47), with *p* = 0.0001. The EG showed a significant improvement in addiction reduction (32.19) compared to the CG (51.93), with *p* = 0.001. The EG demonstrated a significant improvement in attention problems (53.25) compared to the CG (64.33), with *p* = 0.003. The EG significantly improved in reducing aggressive behavior problems (52.69), whereas the CG remained higher (59.80), with *p* = 0.005. The EG showed a significant improvement in engagement in activities (39.56) compared to the CG (29.64), with *p* = 0.001. The EG significantly improved in self-devaluation (42.40), while the CG showed no major changes (57.00), with *p* = 0.044. The EG exhibited a significant improvement in mood repair (28.80), whereas the CG remained nearly unchanged (23.20), with *p* = 0.045. The EG showed a significant improvement in CGI (18.5) compared to the CG (10), with *p* = 0.001. The EG significantly improved in the global functioning assessment (10), while the CG had a lower score (7), with *p* = 0.001. The PIPATIC program demonstrated superiority in the development of a therapeutic alliance compared to cognitive behavioral therapy and in the outcomes of therapeutic change, with *p* < 0.0001 across all compared subscales.	9/9
(L. Wu et al., 2021).	Design: Randomized controlled trial Objective: Investigate the effects of transcranial direct current stimulation (tDCS) on gaming cravings in individuals with Internet Gaming Disorder (IGD).	N = 33 Sex: Male Age: 18–25 years	Internet Gaming Disorder: IAT Craving for Gaming: Cue reactivity task + 9-point scale (1 = “no craving at all,” 9 = “high craving”) Inhibitory control over distractors: Letter categorization area Adverse effects of tDCS: Likert scale for perceived intensity of side effects Perceived differences between active and sham tDCS sessions: Open-ended response	In the active condition, baseline craving significantly decreased after stimulation (from 6.21 to 4.24; *p* < 0.001). In the sham condition, no significant changes were observed in baseline craving (5.94 vs. 5.85; *p* > 0.1). No significant differences were found in cue-induced craving ratings between the active and sham conditions. No clinically significant adverse effects were reported. Participants were unable to reliably distinguish between the active and sham conditions (only 21% attempted, result not different from chance). Active tDCS significantly improved inhibitory control over addiction-related distractors (*p* = 0.004).	11/13
(Nielsen et al., 2021)	Design: Randomized controlled trial Objective: To evaluate the impact of two family therapies—Multidimensional Family Therapy (MDFT) and Traditional Family Therapy (TFT)—on the prevalence and symptoms of Internet Gaming Disorder (IGD).	N = 42 Age = 12–19 years Sex = Male and female (97.6% male)	Internet Gaming Disorder (IGD): Petry IGD Criteria (DSM-5) Gaming Frequency: Timeline Followback Method (TLFB) Quality of Life: Addiction Severity Index (ASC T-ASI) Treatment Satisfaction: Satisfaction Questionnaires	At the 6-month follow-up, the completion rate was 100% in the MDFT group and 86.7% in the FTAU group. At 12 months, 91.7% of adolescents in the MDFT group and 70.0% in the FTAU group completed the follow-up. Regarding the reduction in IGD symptoms, at 6 months, the number of IGD criteria significantly decreased in the MDFT group (83.3%) compared to the FTAU group (60.3%) (*p* < 0.01). At 12 months, the reduction remained greater in the MDFT group (87.9% vs. 63.3%) (*p* < 0.01). At the 6-month follow-up, no adolescents in the MDFT group met IGD criteria, whereas 11.5% of those in the FTAU group still did. At 12 months, IGD was present in 19.0% of the MDFT group compared to 63.3% in the FTAU group (*p* < 0.01). Although IGD criteria significantly decreased in both groups, the reduction was more pronounced in the MDFT group. For instance, “continued gaming despite problems” showed a significantly greater improvement in MDFT compared to FTAU (*p* < 0.01). Gaming time decreased in both groups at 6 months (MDFT: 2.6 hours, FTAU: 3.1 hours); however, at 12 months, gaming time rebounded in both groups, with no significant difference between them (*p* > 0.05). Regarding quality of life, both groups showed significant improvements, but the MDFT group demonstrated a greater reduction in gaming-related problems compared to FTAU (MDFT: −60.9% vs. FTAU: −41.2%) (*p* < 0.05). At 12 months, parents of adolescents in the MDFT group reported a more significant reduction in gaming-related problems (MDFT: −46.8% vs. FTAU: −36.2%) (*p* < 0.05). The MDFT group had a 100% treatment completion rate, while the FTAU group had a significantly lower compliance rate (70%) (*p* < 0.05). The MDFT group attended more treatment sessions (26.33 vs. 17.50 in FTAU), with significantly longer session durations (MDFT: 85.8 min vs. 65.4 min in FTAU) (*p* < 0.01). The number of family therapy sessions was the only factor significantly correlated with a reduction in IGD symptoms at 12 months (*p* < 0.01).	11/13
(Igarashi et al., 2024)	Design: Non-randomized experimental study Objective: To examine the efficacy of a brief mindfulness-based virtual reality intervention on IGD symptomatology.	N = 27 Sex: Men and women Mean age: 20.33 ± 1.12	Internet Gaming Disorder: IGDT-10 and IAT Perceived Stress: PSS-10 Positive and Negative Affect: PANAS-SF	For the IGD group, gaming time had a significant effect on IGD symptoms (*p* = 0.029), indicating that the intervention had a positive impact on IGD symptoms. No significant effect of time on IGD symptoms was found in the control groups (*p* = 0.168). The IGD group showed a significant improvement in IGD symptoms after the 4-week mindfulness-based virtual reality intervention (*p* = 0.014). A significant decrease in weekend gaming time was observed in the IGD group (*p* = 0.034), but no significant change was found in weekday gaming time (*p* = 0.334). After treatment, correlations in the IGD group showed no significant relationships between IGD symptoms and other variables such as perceived stress (PSS), positive affect (PANAS-P), negative affect (PANAS-N), internet addiction (IAT), or gaming time during the week and weekend.	8/9
(Marcelle et al., 2019)	Design: Non-randomized experimental study Objective: To develop and evaluate a multimodal therapy model for Internet Gaming Disorder that includes all relevant psychological factors associated with the disorder.	N = 33 Age: 18–30 years Sex: Men and women	Internet Gaming Disorder: IGD-20 and IAT Quality of Life: WHOQOL-BREF (World Health Organization Quality of Life—Brief Version) Personality: MINI-IPIP (MINI International Personality Item Pool)	A significant difference was found in pre- and post-intervention scores, with a reduction of 10.09 points (*p* < 0.05) in internet addiction. A significant improvement was observed, with a decrease of 9.09 points (*p* < 0.05) in Internet Gaming Disorder. Quality of life dimensions, including the physical domain (*p* < 0.001), psychological domain (*p* = 0.001), and social relationships domain (*p* < 0.001), showed significant improvements after the intervention. However, the environmental domain did not show a statistically significant improvement (*p* = 0.379).	8/9
(Zhang et al., 2016)	Design: Non-randomized experimental study Objective: To examine the effects of a Craving Behavioral Intervention (CBI), developed to reduce gaming-related craving.	N = 59 Sex: Men	Internet Gaming Disorder: CIAS (Chen Internet Addiction Scale) Depression: BDI (Beck Depression Inventory) Anxiety: BAI (Beck Anxiety Inventory) Nicotine Dependence: Fagerstrom Test Hazardous Alcohol Consumption: Alcohol Use Disorders Identification Test (AUDIT) Gaming Craving: Cue-reactivity Task (fMRI) Brain Activation: fMRI (functional magnetic resonance imaging) Brain Connectivity: Functional Connectivity Analysis (PPI—Psychophysiological Interaction)	The CBI+ group showed a significant decrease in CIAS scores (t(22) = 9.49, *p* < 0.001), whereas the CBI− group exhibited a smaller reduction (t(16) = 3.16, *p* < 0.001). There was a significant interaction between session and group in weekly gaming duration (*p* = 0.04). The CBI+ group significantly reduced weekly gaming hours (*p* < 0.001), whereas the CBI− group showed a smaller reduction (*p* = 0.007). The CBI+ group experienced a significant reduction in gaming-related anxiety (*p* < 0.001), while the CBI− group also improved, but to a lesser extent (*p* = 0.008). Individuals with IGD showed greater activation in the dorsal striatum (caudate), brainstem, substantia nigra, anterior cingulate cortex, and posterior cingulate cortex (*p* < 0.05), along with reduced activation in the right insula (*p* < 0.05). A significant positive association was found in the middle temporal gyrus (MTG) between brain activation and gaming craving (*p* = 0.035). A significant interaction was observed between group (CBI+ and CBI−) and session in right anterior insula activation (*p* = 0.04 for CBI+, *p* = 0.008 for CBI−). The CBI+ group exhibited significant connectivity between the right insula and the left lingual gyrus and right precuneus (*p* = 0.001, *p* = 0.006), while the CBI− group showed the opposite pattern in connectivity (*p* = 0.005; *p* = 0.01).	8/9
(Zhao et al., 2022)	Design: Randomized trial Objective: To investigate the effects of different recovery-extinction training (RET) intervals on cue-induced craving, neural activity, and behavioral responses in young individuals with Internet Gaming Disorder.	N = 60 Age: 18–23 years; M = 20.56 ± 1.63 Sex: 2 females/22 males	Anxiety: BAI (Beck Anxiety Inventory) Depression: BDI (Beck Depression Inventory) Intelligence: Raven’s Progressive Matrices Personality: TPQ (Tridimensional Personality Questionnaire) Craving for gaming: EAV (gaming desire before and after exposure to a video) Neural activity related to rewards or punishments: MIDT (Monetary Incentive Delay Task) Memory: MST (Modified Sternberg Task) Neural Activity: EEG (Electroencephalography) Physiological Responses: ECG (Electrocardiogram), SCR (Skin Conductance Response)	A significant reduction in gaming craving was observed in the R-10min-E group three months after the intervention compared to the R-6h-E group (*p* = 0.042). The R-10min-E group showed a significantly faster reaction time compared to the R-6h-E group after training (*p* = 0.028). The R-10min-E group exhibited a significant increase (*p* = 0.039) in P3 amplitude evoked by gain cues in the MIDT, whereas the R-6h-E group showed no changes. A significant improvement in functional connectivity was observed in the R-10min-E group compared to the R-6h-E group with gain cues in the MIDT (*p* < 0.05). No significant training effects were found on working memory, as measured by differences in reaction times (RT) and P3 amplitudes evoked by intrusion cues or new cues (*p* = 0.476).	12/13
(Pornnoppadol et al., 2020)	Design: Non-randomized experimental study Objective: To evaluate and compare the effectiveness of four psychosocial interventions for Internet Gaming Disorder (IGD) in adolescents aged 13 to 17 years.	N = 104 Sex: 82 males, 22 females Age: 13–17 years	Internet Gaming Disorder (IGD): GAST (Game Addiction Screening Test) Quality of Life: GAME-Q (Game Addiction Quality of Life Scale) Protective Factors: GAME-P (Game Addiction Protection Scale) Emotional and Behavioral Symptoms: PSC-17 (Pediatric Symptom Checklist-17)	The severity of Internet Gaming Disorder (IGD) significantly decreased in all intervention groups compared to the control group (*p* ≤ 0.001, 0.002, and 0.005 at 1, 3, and 6 months, respectively). Adolescents who participated in the Siriraj Therapeutic Residential Camp (S-TRC) and the combination of S-TRC and Parent Management Training for Game Addiction (PMT-G) showed a more sustained reduction in online gaming addiction compared to those who received only PMT-G or psychoeducation (*p* ≤ 0.001). Quality of life significantly improved in the intervention groups compared to the control group (*p* ≤ 0.003 at 3 months and *p* ≤ 0.033 at 6 months). Protective factors against addiction significantly increased 3 months post-intervention in the S-TRC and PMT-G groups (*p* ≤ 0.043), although the difference was no longer significant at 6 months (*p* = 0.486). Emotional and behavioral symptoms significantly decreased in the S-TRC and S-TRC + PMT-G groups compared to the other groups (*p* ≤ 0.001 at 3 months). In the control group, most adolescents remained classified as “addicted” or “probably addicted” at 6 months post-intervention, whereas in the S-TRC and PMT-G groups, the percentage of adolescents in these categories was below 50%.	9/9
(Zhang et al., 2018)	Design: Non-randomized experimental study Objective: To investigate how Craving Behavioral Intervention (CBI) affects the functional connectivity of the ventral striatum (VS) circuit in individuals with Internet Gaming Disorder.	N = 115 Sex: Men Age: 18–30 years	Internet Gaming Disorder: CIAS (Chinese Internet Addiction Scale) Anxiety: BAI (Beck Anxiety Inventory) Depression: BDI (Beck Depression Inventory) Impulsivity: BIS-11 (Barratt Impulsiveness Scale—Version 11) Craving for Gaming: QSU-Brief (Adapted version of the Questionnaire of Smoking Urges) Brain Activity: fMRI (Functional Magnetic Resonance Imaging)	Individuals with Internet Gaming Disorder (IGD) exhibited significantly higher resting-state functional connectivity between the ventral striatum (VS) and the left inferior parietal lobule (lIPL), left middle frontal gyrus (lMFG), and right inferior frontal gyrus (rIFG) compared to the healthy control group (*p* < 0.01). Connectivity between the vs. and lIPL showed a significant positive correlation with IGD severity (CIAS) (r = 0.31, *p* = 0.01) and impulsivity levels (BIS-11) (r = 0.26, *p* = 0.03). Participants who received Craving Behavioral Intervention (CBI+) showed a significant reduction in IGD severity, as measured by the CIAS (F(1,34) = 26.60, *p* < 0.001, η^2^ = 0.43), as well as a decrease in time spent on online gaming (F(1,34) = 7.07, *p* = 0.012, η^2^ = 0.17). Functional connectivity between the vs. and lIPL significantly decreased following the intervention in the CBI+ group (F(1,34) = 13.89, *p* = 0.001, η^2^ = 0.29), whereas no changes were observed in the non-intervention group (CBI−).	8/9
(Kang et al., 2018)	Design: Non-randomized experimental study Objective: To investigate the effect of equine-assisted activities and therapies (EAAT) on the affective network of adolescents with Internet Gaming Disorder (IGD).	N = 30 Sex: Male Mean Age: 15.6 years	Internet Gaming Disorder: YIAS (Young Internet Addiction Scale) Attachment: K-ECRS (Korean Experiences in Close Relationships Scales—Revised) Depression: CDI (Children’s Depression Inventory) ADHD Symptoms: K-ARS (Korean ADHD Rating Scale) Functional Connectivity in the Affective Network: Resting-State fMRI (rs-fMRI)	After 7 days of EAAT, adolescents with IGD showed a significant improvement in avoidant attachment, with a reduction in K-ECRS scores (*p* < 0.01) compared to the control group. Video game addiction symptoms significantly decreased in the IGD group (*p* < 0.01), while no relevant changes were observed in the control group. Depressive symptoms improved in the IGD group following EAAT intervention (*p* < 0.01). Functional MRI analysis revealed an increase in functional connectivity within the affective network, specifically from the left amygdala to the medial frontal gyrus (BA 25), which correlated with improvements in avoidant attachment (r = −0.56, *p* = 0.001). In the IGD group, functional connectivity between the left amygdala and the left orbitofrontal gyrus (BA 11) significantly increased after EAAT (*p* < 0.001), suggesting a potential improvement in emotional regulation.	9/9
(J. Han et al., 2020)	Design: Non-randomized experimental study Objective: To evaluate the efficacy of cognitive behavioral therapy (CBT) in the treatment of Internet Gaming Disorder (IGD).	N = 205 Sex: Male Age: 25.9 years	Internet Gaming Disorder: YIAS (Young Internet Addiction Scale) ADHD Symptoms: K-ADHD (Korean ADHD Rating Scale) Depression: BDI (Beck Depression Inventory) Anxiety: BAI (Beck Anxiety Inventory) Impulsivity: BIS/BAS (Behavioral Inhibition System/Behavioral Activation System Scale) Social Avoidance: SADS (Social Avoidance and Distress Scale) Family Cohesion: FES (Family Environment Scale) Clinical Improvement: CGI-I (Clinical Global Impressions—Improvement Scale)	Cognitive behavioral therapy (CBT) was more effective than supportive therapy in improving symptoms of Internet Gaming Disorder (IGD), with a 66.3% improvement in the CBT group vs. 49.0% in the supportive therapy group (χ^2^ = 6.25, *p* = 0.02). In the CBT group, patients showed significant reductions in IGD severity (YIAS: F = 11.76, *p* = 0.001), anxiety (BAI: F = 9.06, *p* = 0.003), impulsivity (BIS/BAS: F = 8.84, *p* = 0.003), and social avoidance (SADS: F = 9.90, *p* = 0.002) compared to the supportive therapy group. Among patients who improved in the CBT group, significant reductions were observed in IGD severity (YIAS: F = 52.48, *p* < 0.001), ADHD symptoms (F = 16.85, *p* < 0.001), depression (BDI: F = 25.11, *p* < 0.001), anxiety (BAI: F = 9.41, *p* = 0.003), impulsivity (BIS/BAS: F = 7.48, *p* = 0.004), social avoidance (SADS: F = 35.19, *p* < 0.001), and family cohesion (FES: F = 9.71, *p* = 0.002). In the supportive therapy group, significant improvements were only observed in IGD severity (YIAS: F = 61.05, *p* < 0.001), ADHD symptoms (F = 28.55, *p* < 0.001), depression (BDI: F = 23.83, *p* < 0.001), and impulsivity (BIS/BAS: F = 10.02, *p* = 0.002), but not in anxiety (*p* = 0.793), social avoidance (*p* = 0.961), or family cohesion (*p* = 0.491). Predictors of better response to CBT included lower ADHD scores (*p* < 0.01), lower depression levels (*p* < 0.05), and not using medication (*p* < 0.01).	9/9
(Li et al., 2018)	Design: Non-randomized experimental study Objective: To investigate the therapeutic effectiveness of the Mindfulness-Oriented Recovery Enhancement (MORE) intervention in reducing Internet Gaming Disorder (IGD).	N = 30 Sex: Male (80%), Female (16.7%), Non-binary (3.3%) Mean Age: 25 years	Internet Gaming Disorder: DSM-5 Criteria Craving: VAS (Visual Analog Scale) Dysfunctional Beliefs About Gaming: Negative Thoughts and Feelings Subscale of the Online Cognition Scale Positive Reappraisal: Cognitive Emotion Regulation Questionnaire (CERQ)	The treatment with MORE significantly reduced dysfunctional cognitions about video games compared to the control group (SG) (*p* = 0.003). Post-treatment dysfunctional cognitions significantly predicted IGD severity at the 3-month follow-up (*p* < 0.001). MORE had a significant indirect effect on reducing IGD criteria through the reduction in dysfunctional cognitions (b = −0.27, 95% CI [−0.53, −0.06]). Craving for video games significantly decreased after the MORE treatment (*p* = 0.001), mediated by the reduction in dysfunctional cognitions (b = −0.20, 95% CI [−0.42, −0.04]). Positive reappraisal significantly increased in the MORE group, but it did not directly predict reductions in IGD or craving (*p* = 0.12 and *p* = 0.07, respectively).	9/9
(Sakuma et al., 2017)	Design: Non-randomized experimental study Objective: To evaluate the efficacy of the Self-Discovery Camp (SDiC), a Japanese residential therapeutic camp, in improving the symptoms of Internet Gaming Disorder (IGD).	N = 10 Sex: Male Mean Age: 16.2 years	Internet Gaming Disorder: Hours per day/week (Questionnaire + Interview) Problem Recognition: SOCRATES (Stages of Change Readiness and Treatment Eagerness Scale)	The total internet gaming time significantly decreased three months after treatment at the Self-Discovery Camp, with an average reduction of 3.18 h per day (*p* = 0.044, d = 1.15). The number of hours spent gaming per week significantly decreased, with a reduction of 27.04 hours per week (*p* = 0.024, d = 1.26). The number of days per week participants played did not show significant changes post-treatment (*p* = 0.18, d = 0.31). Self-efficacy significantly improved, with an increase in the “Taking Action” subscale score of the SOCRATES scale (*p* = 0.012, d = 1.00), indicating greater confidence among participants in reducing their addictive behavior. No significant differences were found in the “Problem Recognition” subscale (*p* = 0.45) or the “Ambivalence” subscale (*p* = 0.28), suggesting that the perception of the disorder did not change substantially. A significant positive correlation was found between the age of onset of IGD and problem recognition (r = 0.781, *p* = 0.008), indicating that participants who developed the disorder at an older age were more aware of their addiction.	8/9
(Yao et al., 2017)	Desing: Experimental Study non-randomized Objective: Valuate the efficacy of a group behavioral intervention combining reality therapy and mindfulness meditation in reducing decisional impulsivity and IGD severity.	N = 46 Sex: Not specified Age: 18–26 years	Internet Gaming Disorder: CIAS (Chinese Internet Addiction Scale) Impulsivity in Decision-Making: DDT (Decision Delay Task), BART (Balloon Analog Risk Task) Anxiety: BAI (Beck Anxiety Inventory) Depression: BDI (Beck Depression Inventory)	The IGD and HC groups did not differ significantly in age or years of education (*p* > 0.05). The IGD group scored significantly higher than the HC group on CIAS, BAI, and BDI (*p*s < 0.001), with effects remaining significant after Bonferroni correction (*p* < 0.01). In the DDT, the IGD group showed higher impulsivity in decision-making, reflected in a higher log-transformed k value compared to the HC group (*p* = 0.001). In the BART, the IGD group exploded more balloons than the HC group (*p* = 0.023), but no significant differences were found in the adjusted average number of pumps. After the intervention, the IGD group showed a significant reduction in the k value on the DDT compared to baseline measurement (F(1,35) = 15.90, *p* < 0.001, η^2^ = 0.31), whereas the HC group showed no significant changes (F(1,35) = 0.06, *p* = 0.81). The IGD group experienced a significant decrease in CIAS, BAI, and BDI scores after the intervention (*p*s < 0.001). In the multiple linear regression, only changes in the k value on the DDT were significantly associated with changes in CIAS scores (Beta = 0.53, *p* = 0.04).	8/9
